# Supplementary material for: The causal relationship between the human gut microbiota and pyogenic arthritis: a Mendelian randomization study
Source: Front Cell Infect Microbiol. 2024 Nov 26;14:1452480. doi: 10.3389/fcimb.2024.1452480 (PMC11629706; doi:10.3389/fcimb.2024.1452480)
Supplement: Supplementary file 1 [file DataSheet1.zip › mendelian test/ebi-a-GCST90027718.csv_leave_one_out.pdf]

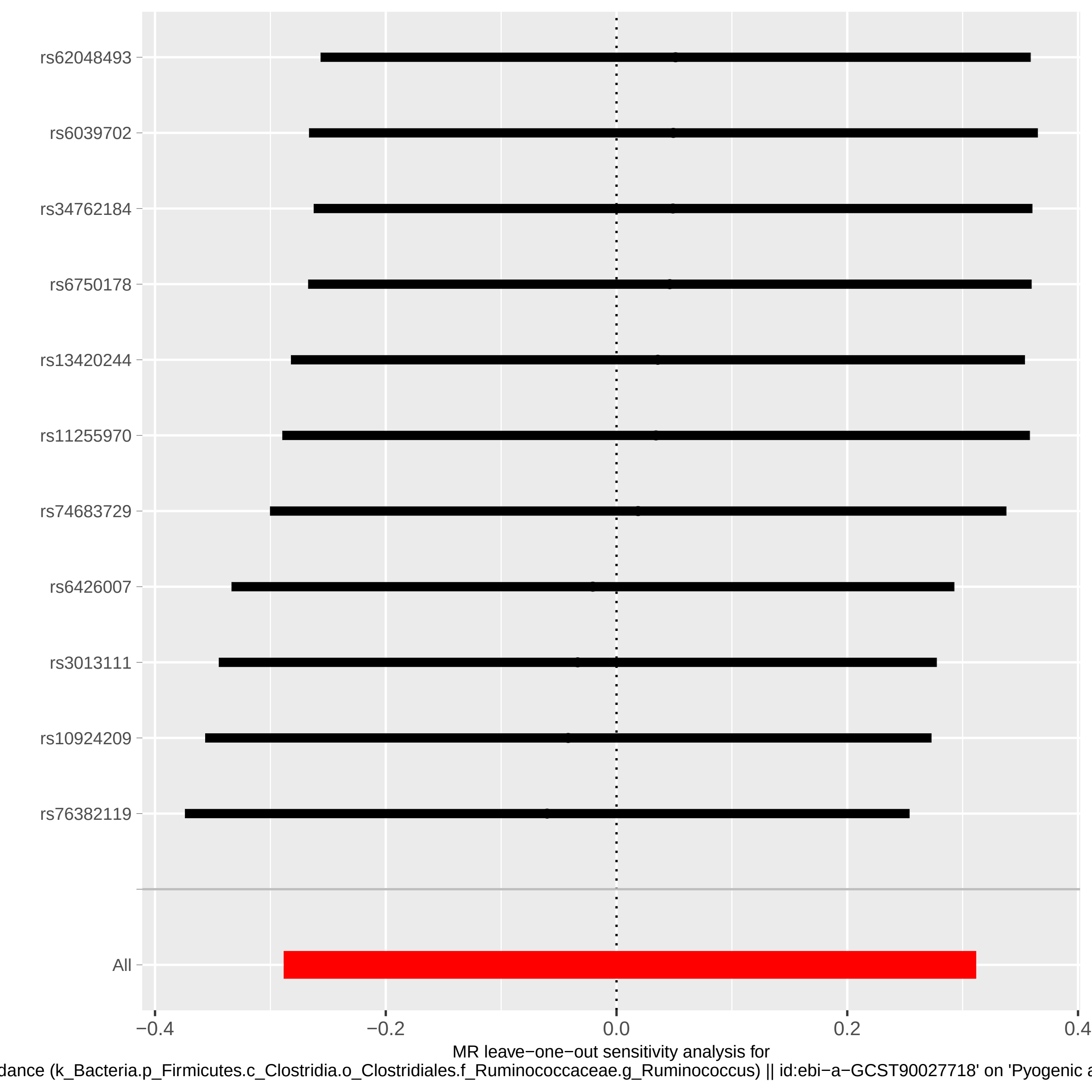

rs62048493
rs6039702
rs34762184
rs6750178
rs13420244
rs11255970
rs74683729
rs6426007
rs3013111
rs10924209
rs76382119
All
−0.4
−0.2
0.0
0.2
0.4
MR leave−one−out sensitivity analysis for
dance (k_Bacteria.p_Firmicutes.c_Clostridia.o_Clostridiales.f_Ruminococcaceae.g_Ruminococcus) || id:ebi−a−GCST90027718' on 'Pyogenic a
